# Soundscape in city and built environment: current developments and design potentials

**DOI:** 10.1007/s44213-022-00005-6

**Published:** 2023-01-12

**Authors:** Jian Kang

**Affiliations:** UCL Institute for Environmental Design and Engineering, The Bartlett, University College London, London, WC1H 0NN UK

**Keywords:** Soundscape, Noise, Design, Planning, Open public space, Built environment

## Abstract

In the field of environmental acoustics, the conventional approach of reducing ‘sound level’ does not always deliver the required improvements in quality of life. Soundscape, defined by the ISO as the ‘acoustic environment as perceived or experienced and/or understood by a person or people, in context’, promotes a holistic approach, regarding sounds as ‘resources’ rather than just ‘wastes’. The first part of this review/position paper, mainly using the works by the author and the teams/collaborators as examples, discusses the current developments in soundscape, in terms of soundscape understating and exchanging, collecting and documenting, harmonising and standardising, creating and designing, and outreaching, showing that while considerable works have been carried out, much work is still needed, in terms of basic research, and more importantly, research towards practice. The second part of this paper then explores a soundscape approach in the urban sound design/planning process. With a proposed framework for designing soundscape in urban open public spaces, considering four key components, including characteristics of each sound source, acoustic effects of the space, social/demographic aspect of the users, and other physical conditions, design potentials are demonstrated.

## Introduction

Environmental noise is often the main cause of environmental distress in terms of the number of complaints received (Tong et al. 2021). The EU Directive Relating to the Assessment and Management of Environmental Noise (END) has led to a number of major actions (EU 2002), where reducing noise level has been the focus. However, such a conventional approach does not always deliver the required improvements in quality of life, as the noise annoyance by inhabitants only depends on approximately 20–40% of the acoustic parameters (Job 1988; Guski 1998; Berglund 1998; Lercher 1998). For example, in urban open public spaces it has been shown that when the sound level is below a certain value, as high as 65-70dBA, people’s acoustic comfort evaluation is not well related to the sound level, whereas the sound type, the user characteristics and other factors play an important role (Yang and Kang 2005a, b; Kang 2006). With the development of electric vehicles, the sound environments will become quieter. However, it is noted that during the COVID-19 lock-down, although the environmental noise levels were much lower (Aletta et al. 2020), there was a sharp increase in noise complaints, as some other sound sources became more annoying even their sound levels were unchanged (Tong et al. 2021).

The soundscape strategy, by integrating both wanted and unwanted sounds and considering sound environment as perceived, in context, with an interdisciplinary approach (International Organization for Standardization 2014), is a growing field for addressing this gap (Kang and Schulte-Fortkamp 2016). Although the term soundscape was introduced in the 1960s (Schafer 1977), significant attention to it has only been paid by researchers and practitioners with the END actions on creating/protecting quiet areas as a main driver. In the last 20 years much work has been carried out in soundscape, bringing a step change in the field of environmental acoustics, beyond noise control engineering.

This review/position paper first discusses the current developments in soundscape in various facets of soundscape, from basic research, to practice, to outreaching, mainly using the works by the author and the teams/collaborators as examples (Kang 2007, 2008, 2010a, b, 2017, 2019, 2021; Kang et al. 2016), based on a number of conference keynote presentations by the author. Then it explores a soundscape approach in the urban sound design/planning process (Kang 2007, 2010a, b, 2017, 2018). With a proposed framework for designing soundscape in urban open public spaces, the design potentials of the four key components, including sound sources, space, people, and environment, are demonstrated (Kang et al. 2016; Zhang and Kang 2007).

## Current developments in soundscape

The pioneering works in soundscape were carried out by Schafer (1977), on relationships between the ear, human beings, sound environments and society. In 1975, Schafer led a group on a European tour of five villages where they made detailed investigations of the soundscape. Later the five villages were revisited to undertake comparative studies, to analyse how their soundscapes had changed due to urbanisation (Järviluoma 2000; Järviluoma et al. 2010). The World Forum for Acoustic Ecology (WFAE) was founded in 1993, with members who share a common concern with the state of the world soundscape as an ecologically balanced entity. While the WFAE represents a multi-disciplinary spectrum of individuals engaged in the study of the social, cultural, and ecological aspects of the sonic environment, less attention has been paid to the planning, designing and engineering the soundscapes of built environments.

In 2002, the publication of the END (EU 2002) led to a number of major actions, including identifying/preserving quiet areas (Shepherd and Grimwood 2009). While it was not clear how to identify those quiet areas, where to go with it, how to use it, or how to incorporate it in design, a step change was needed, by developing a new method to assess sound environment quality. Correspondingly, ISO/TC43/SC1/Working Group 54 was formed to work on ‘Perceptual assessment of soundscape quality’. ‘Soundscape’ is different from ‘acoustic environment’ as it relates to perceptual constructs rather than just physical phenomena. It promotes a holistic approach, regarding sounds as ‘resources’ rather than just ‘wastes’, and focuses on “wanted” (preference) rather than just “unwanted” (discomfort) sounds.

The impacts of soundscape therefore include: (1) Health: It can help to provide supportive environments which prevent the degradation of functional health, and enhance the engagement in health promoting activities less likely in unpleasant neighbourhoods. (2) Culture: It is important in terms of ‘sensing of places’, tourism, and conservation. (3) Economy: It can bring prosperity to the economy, i.e. property prices, offset health costs through provision of restorative living spaces, reduce costs caused by anti-social behaviours, and prevent unnecessary noise mitigating actions (Kang et al. 2013; Kang and Schulte-Fortkamp 2016).

**Fig. 1:**
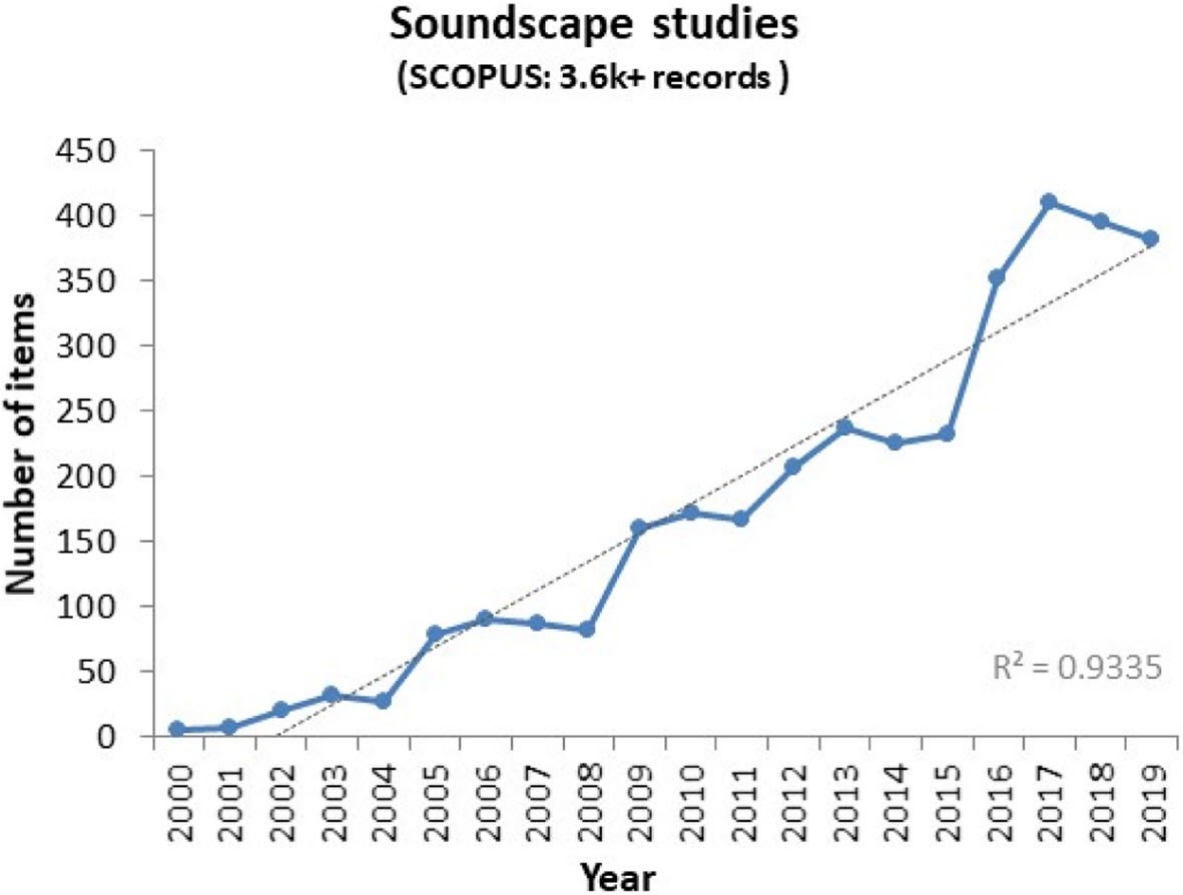
Number of soundscape studies published since 2000 as covered in Scopus database. *-TITLE-ABS-KEY(soundscape) AND (LIMIT-TO (SUBJAREA,"PHYS”) OR LIMIT-TO (SUBJAREA,"ARTS”) OR LIMIT-TO (SUBJAREA,"SOCI”) OR LIMIT-TO (SUBJAREA,"COMP”) OR LIMIT-TO (SUBJAREA,"ENVI”) OR LIMIT-TO (SUBJAREA,"ENGI”) OR LIMIT-TO (SUBJAREA,"EART”) OR LIMIT-TO (SUBJAREA,"PSYC”) OR LIMIT-TO (SUBJAREA,"MULT”) OR LIMIT-TO (SUBJAREA,"NEUR”) OR LIMIT-TO (SUBJAREA,"ENER”) OR LIMIT-TO (SUBJAREA,"BUSI”) OR LIMIT-TO (SUBJAREA,"DECI”) OR LIMIT-TO (SUBJAREA,"HEAL”) OR LIMIT-TO (SUBJAREA,"NURS”) OR LIMIT-TO (SUBJAREA,"ECON”)) AND (LIMIT-TO (PUBYEAR,2000–2020)*

Soundscape activities have since increased significantly, as reflected in the number of publications shown in Fig. 1 (Kang 2021). There have been increasing special sessions in conferences in the field of acoustics such as International Congress and Exposition on Noise Control Engineering (Internoise), European Congress and Exposition on Noise Control Engineering (euronoise), International Congress on Acoustics (ICA), International Congress on Sound and Vibration (ICSV), International Commission on Biological Effects of Noise (ICBEN), and Western Pacific Acoustics Conference (WESPAC), and also in the field of planning such as Association of European Schools of Planning (AESOP) Conference. There have also been increasing national and international research projects, practical projects (Kang et al. 2013), as well as networks such as the EU-COST network on Soundscape of European Cities and Landscapes (Kang et al. 2013), with partner organisations from 23 COST countries and 7 outside Europe, covering a range of disciplines in science, engineering, social science, humanity and medicine, and the Global Sustainable Soundscape Network GSSN.

**Fig. 2:**
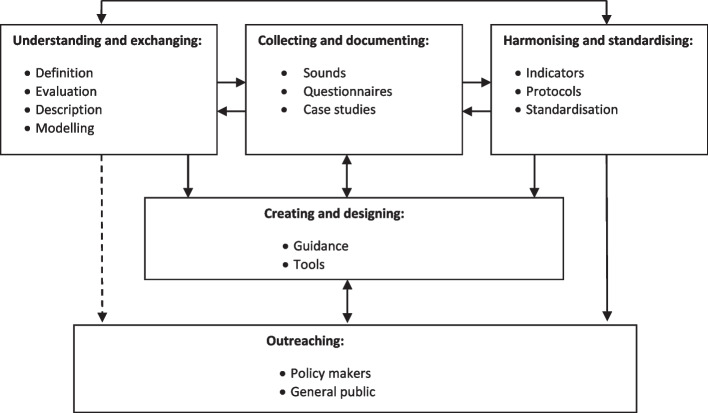
A framework for soundscape research and practice based on EU-COST network on soundscape (Kang et al. 2013)

To review the current developments of soundscape research and practice, a framework based on the EU-COST network on soundscape is used, as shown in Fig. 2 (Kang et al. 2013), where five main issues are considered, including soundscape understating and exchanging, collecting and documenting, harmonising and standardising, creating and designing, and outreaching. In this section, works in those facets are reviewed, mainly using the work from the author and the teams/collaborators as examples.

### Understanding and exchanging

#### Definition

To understand the elements in the perceptual construct of soundscape, the ISO/TC43/SC1/Working Group 54 proposed a framework including interactions among context, sound sources, acoustic environment, auditory sensation, interpretation of auditory sensation, responses, and outcomes (Schomer et al. 2010; International Organization for Standardization 2014). Correspondingly, in ISO 12913-1:2014 Acoustics – Soundscape - Part 1: Definition and conceptual framework, soundscape is defined as the ‘acoustic environment as perceived or experienced and/or understood by a person or people, in context’ (International Organization for Standardization 2014).

#### Evaluation

In terms of psychological or subjective evaluation of soundscape, a large amount of investigations have been carried out, considering (1) a range of spaces and functions including urban streets, urban open public spaces, parks, schools, bus stations, theme streets, cycle paths, outdoor concerts, racing tracks, archaeological sites, covered spaces, underground shopping streets; (2) a range of sound sources, from noise sources including industrial noises, aircraft noises, road noises, wind turbines, amplified music; to positive sounds including natural sounds; to sources with mixed perceptions such as an infant’s cry; and (3) a range of users, where their social, demographical and cultural characteristics have been considered for various users, also for specific groups such as children, deaf, hearing impaired and blind people (Kang 2006, 2007; Kang and Schulte-Fortkamp 2016; Yu and Kang 2014; Torresin et al. 2020).

In terms of the physiological side of soundscape, there has been relatively limited, but increasing work, considering a range of indicators including heart rate, respiratory rate and forehead electromyography levels, and using a range of tools including fMRI techniques (Watts et al. 2009; Irwn et al. 2009; Hunter et al. 2010; Medvedev et al. 2015; Li and Kang 2019). Some initial relations have been established between psychological, physiological, and health indicators (Hume and Ahtamad 2009; Aletta et al. 2018; Erfanian et al. 2019).

A wide range of perspectives have been explored in soundscape, including how attention on the external environment is shaped internally; interactions between emotion and soundscape; how expectation affects soundscape; interactions between behaviour and soundscape; how social relations, integration and support affect soundscape; how meaning is attached to the objects within a cultural/societal context; the effect that a stressed or harmonised human-environment relationship can have on mental health; and soundscape valuation, following noise valuation (Ge and Hokao 2004; Dubois and Catherine 2007; Aletta et al. 2016; Ren et al. 2018a; Cao and Kang 2019; Qin et al. 2020; Jiang et al. 2022).

Multi-sensory interactions have also been explored and demonstrated, including audio-visual interactions, sound-smell interactions, and sound-thermal interactions (Ren and Kang 2015; Ba and Kang 2019a, b; Jin et al. 2020a, b). Of various physical conditions the aural-visual interactions have been intensively studied. Significant correlations have been found between landscape and acoustic satisfaction, between visual and acoustic satisfaction, as well as between view and quietness in choosing a living environment (Kang 2006; Liu et al. 2022). Aural-visual interactions have also been proved physiologically (Hunter et al. 2010).

#### Description

Based on the above evaluation work, determining essential factors and framework for soundscape description is important for understanding as well as creating/designing soundscapes. A framework of designable factors for soundscape in urban open public spaces has been developed, as further discussed in Section 3 of this paper (Zhang and Kang 2007). A taxonomy has also been developed showing categories of places, sounds, and sound sources (Brown et al. 2011).

#### Modelling

It is important to integrate the knowledge acquired from different fields into explicit modelling. Using the data obtained from a large-scale survey, a model based on artificial neural networks has been developed to predict soundscape perception (Yu and Kang 2009). More fundamentally, a bottom-up approach is also needed, considering the individual sensory, cognitive and emotional mechanisms (De Coensel and Botteldooren 2008; Niessen et al. 2009). Modelling physics side, namely sound propagation is a space is also relevant here, where a number of models have been developed (Kang 2005; Attenborough et al. 2006). Finally, for practical applications of those models, applicability is of great importance, considering different sectors such as planning, designing, management, and engineering, and different scales, including macro-scale (i.e. a city), meso scale (i.e. a residential block) and micro-scale (i.e. a street or square).

### Collecting and documenting

Gathering and maintaining a repository of soundscape data is important, to be achieved and re-analysed and studied from inter- and trans- disciplinary perspectives. In terms of sound sources, there have been a considerable number of databases of various kinds, using different recording techniques. While those are useful for soundscape studies, more relevant databases would be those with context information, such as visual information. However, such databases are still limited although in recent years there is an increasing number, such as those based on the Urban Soundscapes of the World project (2017), European Research Council (ERC) Soundscape Indices (SSID) project (Kang et al. 2019), and SONYC – Sounds of New York City project (2014).

Given that a large number of soundscape questionnaire surveys and interviews have been carried out (Kang et al. 2013), such as those based on RUROS (Yang and Kang 2005a, b; Yu and Kang 2008) and SSID projects (Kang et al. 2019), with 10,000 and 4000 field surveys/interviews worldwide respectively, there is a recognised need to create coordinated and comparable databases.

To move from research to practice, a collection of good soundscape design examples and case studies is vital across sectors including researchers, practitioners and policy makers. There have been some initial efforts (Kang and Schulte-Fortkamp 2016) and a more systematic collection project is on-going through the Catalogue of Soundscape Intervention project (2021).

### Harmonising and standardising

While it is argued that standardisation could restrict the creativity in designing soundscapes, for example, in terms of the scopes (i.e. practical work vs creative arts and soundscape compositions) and evaluation methods (i.e. by designers vs users), from planning viewpoint, it is useful to have standards. The ISO/TC43/SC1/Working Group 54 has generated a series of standards and technical specifications on perceptual assessment of soundscape quality, including ISO 12913-1: 2014: Part 1: Definition and conceptual framework (International Organization for Standardization 2014), ISO/TS 12913-2: 2018: Part 2: Data collection and reporting requirement (International Organization for Standardization 2018), and ISO/TS 12913-3: 2019: Part 3: Data analysis (International Organization for Standardization 2019). Currently the Working Group is developing ISO/TS 12913-4 - Part 4: Design and intervention.

Correspondingly, a number of more detailed and specific protocols have been developed, such as those for soundscape description and evaluation, considering cross-cultural and cross-contextual differences; and measurement procedures with respect to a balance between scientific accuracy and practical applicability, also considering comparability and reproducibility. A recent example is the SSID protocol (Mitchell et al. 2020).

There is still a recognised need to develop a new set of indicators to characterise sound quality of environments that improves significantly on the conventional decibel level approach that has been the basis of current regulations worldwide for decades. The indicators should be suitable to assess health related quality of life and functional health which can then be used to evaluate claims related to health-promotion benefits. Efforts have been made to develop indicators including fuzzy noise limits, tranquillity rating, speech intelligibility, similarity index, and hierarchical clustering (Raimbault et al. 2001; Hiramatsu et al. 2001; Licitra and Memoli 2005; Pheasant et al. 2009; Davies et al. 2009; Woloszyn et al. 2009; Kang 2007, 2010a, b, 2017).

An ongoing ERC Advanced Grant project aims to establish “soundscape indices” (SSID) (Kang et al. 2019). By taking psychological, (psycho)acoustical, neural and physiological, and contextual factors into account, SSID will adequately reflect levels of human comfort, to integrate side-by-side with (and eventually replace) decibel-based metrics into existing (international) regulations. Steps to achieve this include:

1. to characterise soundscapes, by capturing acoustic environments and establishing a comprehensive database;
2. to identify key factors and their influence on soundscape quality based on the database, by conducting laboratory psychological evaluations, acoustical/psychoacoustic factors analysis, and also, to research the neural and psychophysiological underpinnings of soundscape experience;
3. to develop, test and validate the soundscape indices, by analysing the influences of various factors, and by developing prediction models.

The soundscape indices may take the form of a single index or a set of indices. For the former, it could be SSID = f(physical factors) + f(contextual factors) + …, with corrections by socio-demographical factors and modifications with psychological, neural, and physiological considerations. For the latter, the SSID will reflect multiple attributes, and in the same time, they could also be regarded as intermediate indices.

### Creating and designing

In the process of applying soundscape research into practice, there is a need for practical guidance in soundscape design (Hellström 2009). Such guidance (Kang et al. 2004), which currently is still limited, should include design processes, effectiveness of design changes, relevant technical details, as well as good practical examples. In the design process, a key component is public participation (Xiao et al. 2017). It is also of significance to provide guidelines for the preservation of architectural heritage sites from a soundscape perspective (Jia et al. 2020a, b). In the development of such guidance, different needs should be taken into account, for example, from planners, designers, architects, landscape architects, engineers, policy makers, and operation managers.

Correspondingly, soundscape tools and software are also essential, considering different stages and scales, including planning, designing, and management/operation. A number of mapping tools have been developed, such as soundscape perception prediction model based on ANN (Yu and Kang 2009), sound source mapping tools (Liu et al. 2014; Hao et al. 2015), and psychoacoustic mapping tools (Fiebig and Genuit 2009). A soundscape management model for delivery sound environment has also been developed, where with the input in contextual and acoustic factors, the model can predict soundscape quality (Kang et al. 2015).

Auralisation tools are especially relevant and important for soundscape design and also for public participation. While such tools have been developed well in room acoustics, for environmental soundscapes, there are many challenges, including multiple, complex, and moving sources, under complicated situations, as well as speed needed to generate results. On the other hand, there are relatively low requirements in terms of accuracy compared to room acoustics. Therefore, it is important to explore simplifications through subjective experiments (Smyrnova and Kang 2010; Xu and Kang 2019).

### Outreaching

As discussed above, policies have been a major driver to move from research to practice. For policy makers it is important to demonstrate benefits, especially health effects, and provide successful examples and tools (Kihlman 2007; Kang et al. 2013). With the influence of soundscape work, the UK noise policy (UK DEFRA 2010) has been moved from noise mitigation to noise management, and another step change was that in 2018, the Welsh Government (2018) published its Noise and Soundscape Action Plan. Soundscape is also relevant to a wider range of policy. For example, it should be recognised that soundscape studies are not only for the improvement of the current sound environment but also for the conservation of our sound environments which can be classified as acoustic heritages (Brambilla et al. 2007; Kang et al. 2013; Huang and Kang 2015).

It is equally important to create awareness amongst the general public, especially given that soundscape is relevant to a much wider range of citizens than noise (Kang and Aletta 2020). For that soundscape art installations could be effective (McGinley 2005), as well as apps which involve participation of the general public, such as Noise Capture (2022).

## Design potentials in soundscape

There are great potentials in applying soundscape approaches in a wide range of places. For example, a model has been developed for managing the delivery sounds in London, where the acoustics parameters such as sound level, as well as contextual parameters, have been taken into account (Kang et al. 2015). Conservation is an important application of soundscape too, such as the soundscape conservation in Tibet (Huang and Kang 2015) and Guizhou (Mao et al. 2013), and in religious context (Zhang et al. 2016). Moreover, rural soundscape has been subject to increasing attention (Ren et al. 2018b; Yu and Kang 2018). The soundscape approach has also been applied in changing people’s behaviour, for example, in way-guidance (Aletta et al. 2016). Soundscape could be applied at different scales, from micro-scale such as a square and a street, to meso scale, such as a residential area, to macro-scale, such as a city (Kang et al. 2018; Margaritis and Kang 2017a, 2017b; Margaritis et al. 2018).

**Fig. 3:**
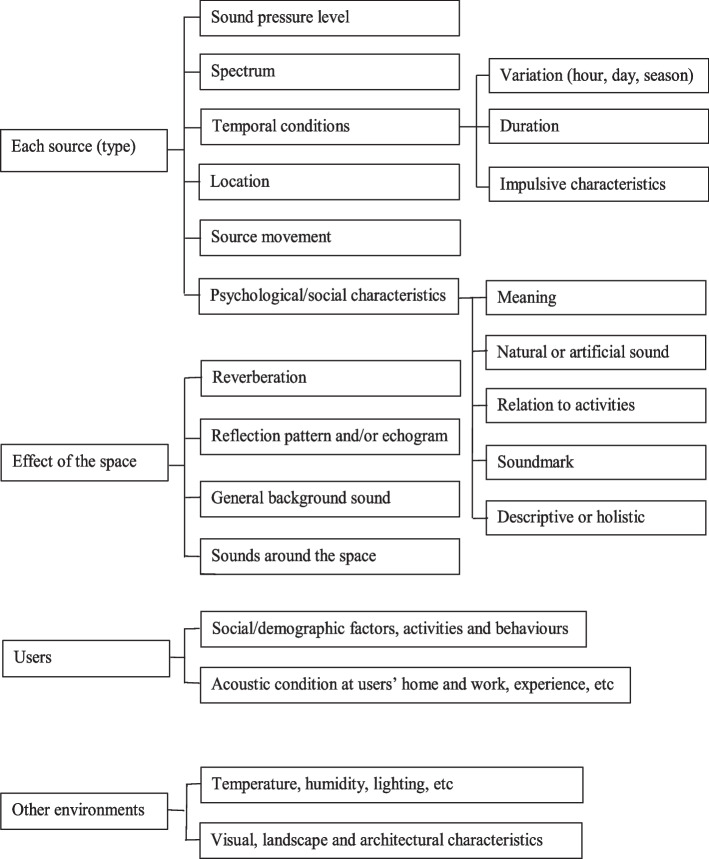
A soundscape design framework for urban open public spaces (Kang 2006)

Urban open public spaces have been extensively examined in term so soundscape, and Fig. 3 shows a soundscape design framework for such spaces (Kang 2006, 2008), where four key components are included: (1) sources - characteristics of each sound source; (2) space - acoustic effects of the space; (3) people – social/demographic aspect of the users as well as their activities and behaviours; and (4) environment - other aspects of the physical environmental conditions. In this section the design potentials are discussed from those four key components. It is noted that the design process is also vital (Xiao et al. 2017), although not discussed in detail in this paper.

### Sound

There is a great potential for planning and designing various sounds, considering the parameters listed in Fig. 3. It would be important to consider soundmarks, reflecting traditional and cultural characteristics, given that the first noticed sounds do not have to be the loudest (Yang and Kang 2005a, b). Spectrum analysis is vital, both for individual sounds and for the overall acoustic environment. The design of soundscape in an urban open public space should also be considered as a dynamic process. From the design viewpoint, preferred sounds in urban public spaces can be divided into sounds from human activities, defined here as ‘active sounds’, and sounds from the landscape elements, for functional and aesthetical purposes, defined here as ‘passive sounds’ (Kang and Yang 2002).

Live music is a typical active sound. An extensive field survey shows that people are not only interested in the music itself, but are also attracted by the activities of the players. In this case, the type of music (e.g. classical music or pop music) is not a very important issue. In terms of spectrum characteristics, case studies in Sheffield suggest that the low frequency components in music are often not loud enough to mask traffic sound, whereas the high frequency components can result in the music emerging over other background sounds, making the soundscape more pleasant. It is important to note that when music is from a store or played through a public address (PA) system, the type of music and the sound level needs to be considered carefully. Most people do not like loud music played from loudspeakers, regardless of the music type (Kang 2007).

**Fig. 4:**
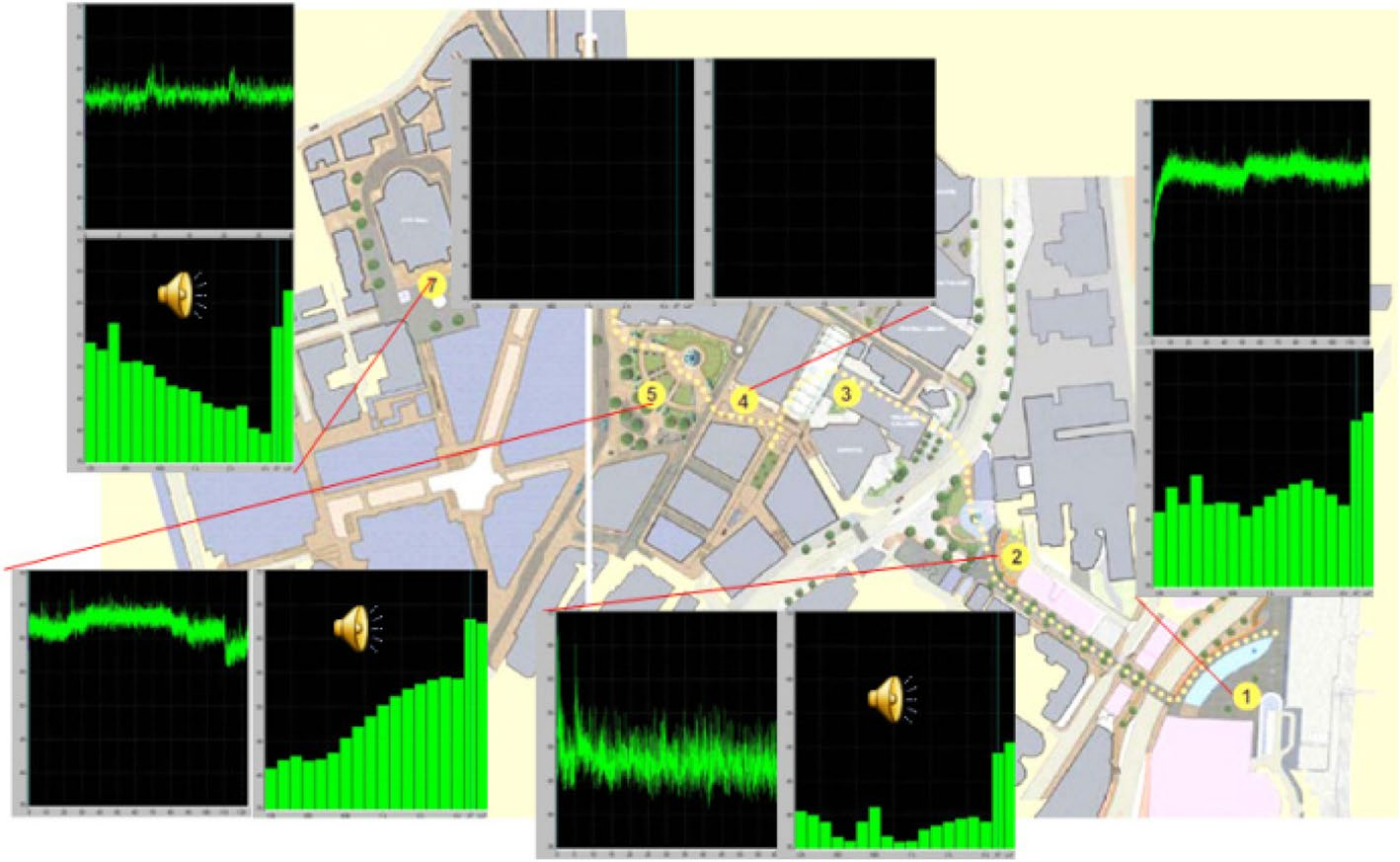
Diversity of water sounds in terms of spectrum and dynamic process, measured at 1 m from each water feature along the Sheffield Gold Route (Kang 2012)

Water is a typical passive sound. In the form of fountains, springs or cascades, it is often used as a landscape element in open public spaces, with endless effects in colouring the soundscape. In the visual aesthetic field, there are contents called ‘primary landscape qualities’, which have a special effect on preference, and water and foliage were two of the contents first identified (Kang and Yang 2002). Similarly, water sounds can be defined as a ‘primary soundscape quality’. Figure 4 shows a wide range of diversity of water sounds in terms of spectrum and dynamic process, measured in the Sheffield Gold Route (Kang 2012). Different flow methods result in different frequencies. Generally speaking, high frequency components come from the water splash itself, whereas when a large flow of water is raised to a significant height and then dropped to a water body or hard surface, notable low frequency components can be generated.

### Space

Simulations of sound propagation were made in a number of hypothetical urban squares surrounded by buildings (Kang 2005) and it has been shown that if a relatively far field is considered, the sound pressure level (SPL) is typically 6-9 dB lower when the square side is doubled; 8 dB lower when the height of buildings surrounding the square is decreased from 50 m to 6 m (diffuse boundaries); 5 dB (diffuse boundaries) or 2 dB (geometrical boundaries) lower if the length/width ratio is increased from 1 to 4; and 10-12 dB lower if the boundary absorption coefficient is increased from 0.1 to 0.9. Similarly, other landscape elements may be effective too, such as vegetation, urban furniture and barriers. Reverberation time (RT) is also an important index for the soundscape in urban open public spaces. Calculation shows that compared to diffusely reflecting boundaries, spaces with geometrically reflecting boundaries have RT and early decay times (EDT) that are significantly longer, typically by 200–400%. Overall, those results suggest that in urban open public spaces, architectural changes and urban design options could affect the sound field significantly (Kang 2006, 2007).

An urban open public space can be designed to encourage activities which generate active soundmarks. For example, a green space may enhance the natural appeal of a public space, attract wild animals’ activities such as bird singing, and improve the microclimate conditions and sound level distribution. Hard spaces are useful for generating many activities, especially for young people, such as dancing and skateboarding. Some patterns of design are more suitable for certain activities, for example, defined edges, such as by walls, colonnades, or shrub plantings, often encourage activities to take place (Kang 2007).

### People

Understanding how soundscape affects its users is a key part of soundscape research. As indicated in Section 2.1, much work has been carried out, both under field and laboratory conditions, considering a range of spaces and locations, sound sources and people (Kang 2010b). Various factors have been examined, including (Kang 2006) (1) social and demographic factors, including age, gender, education, profession, residential status (i.e. local and non-local), cultural background, and acoustic environment at home and at work; (2) activities including moving types such as walking, playing with children, and sport; and non-moving types such as sitting, standing, reading, and watching; and (3) behaviours such as wearing earphones and sunglasses. The results have clearly demonstrated the importance and potential in considering the characteristics of the users. For example, with increasing age, people tend to prefer bird songs, a typical natural sound. In other words, if an urban open public space is mainly designed for older people, more natural sounds like bird songs should be introduced (Kang 2007).

### Environment

The interaction between acoustic and other physical environments is an essential consideration in soundscape planning and design in urban open public spaces. For example, if a place is very hot or very cold, the acoustic comfort could become less critical in the overall comfort evaluation. As mentioned in Section 2.1.2, various interactions have been studied, including between smell and noise, namely with better smell from fragrant trees (Ba and Kang 2019a, b), traffic noise annoyance is reduced; and between thermal comfort and noise annoyance (Jin et al. 2020a, b).

Among various physical conditions the aural-visual interactions have been intensively studied. Based on the data of 14 urban open public spaces in Europe in terms of subjective evaluation of various physical indices, factor analysis shows that visual and auditory aspects are always in the same factor, covering 17–19% of the total variance (Kang 2006). Recently this has also been proved from neural science viewpoint (Hunter et al. 2010). Given these aspects have interactions, working together towards the overall comfort, they should be integrated and optmised in design considerations.

## Discussions

While in Section 2 some immediate needs for developments in soundscape have been mentioned, in the future, there will be a range of major changes in various aspects of our life and environment, so that long-term challenges in soundscape research and practice in such changing situations should be explored (Kang 2021).

New technologies are being developed rapidly and our cities and living environments are becoming smarter, with many devices with sensors rapidly increasing in popularity. This could lead to smarter and adjustable soundscapes, in terms of space and time domain, integrating more the needs of specific users. With the development of electric vehicles, our sound environments might become quieter, which brings more challenges in soundscape creation.

With the tendency of climate change and global warming, there will be a range of changes in our environmental conditions, and this in turn, will bring changes in biodiversity, urban morphology, as well as cultural changes. Those will affect sound sources, sound propagation, and sound preferences. For example, it has been shown that for sounds from water features, surrounding speech, and birdsongs, the sound preferences differ significantly with different climate and culture conditions (Yu and Kang 2014).

Along with the new industrial revolution and climate change, people’s living style will also change. For example, people may work from home more, and use public spaces in a different way. Correspondingly, the soundscape evaluation framework would also change. It has been shown that for indoor soundscape in residential environments, there is a different assessment mode compared to that in urban open public spaces (Torresin et al. 2020).

## Conclusions

Started from the pioneering work in soundscape just over 50 years ago, in the field of environmental acoustics there has been a focus shift from noise control to soundscape creation, and also a shift from soundscape concept to practice. While considerable works have been carried out in terms of soundscape understating and exchanging, collecting and documenting, harmonising and standardising, creating and designing, and outreaching, much work is still needed, in terms of research towards practice, as well as basic research.

Based on the discussions with a soundscape design framework in urban open public spaces, the design potentials of the four key components, namely sounds, space, people, and environment, have been demonstrated. It is expected such a systematic approach towards intentionally designing/planning soundscape will greatly benefit practice and polices, in urban open public spaces and beyond – in different context and at different scales (Kang 2007).

## Data Availability

Not applicable as this is a review paper.

## References

[CR1] Aletta F, Lepore F, Kostara-Konstantinou E, Kang J, Astolfi A (2016). An experimental study on the influence of soundscapes on people’s behaviour in an open public space. Appl Sci.

[CR2] Aletta F, Oberman T, Kang J (2018). Associations between positive health-related effects and soundscapes perceptual constructs: a systematic review. Int J Environ Res Public Health.

[CR3] Aletta F, Oberman T, Mitchell A, Tong H, Kang J (2020). Assessing the changing urban sound environment during the COVID-19 lockdown period using short-term acoustic measurements. Noise Map.

[CR4] Attenborough K, Li KM, Horoshenkov K (2006). Predicting outdoor sound.

[CR5] Ba M, Kang J (2019). Effect of a fragrant tree on the perception of traffic noise. Build Environ.

[CR6] Ba M, Kang J (2019). A laboratory study of the sound-odour interaction in urban environments. Build Environ.

[CR7] Berglund B (1998) Community noise in a public health perspective. In: Proceedings of the 27th international congress and exposition on noise control engineering, vol 1, Christchurch, pp 19–24

[CR8] Brambilla G, De Gregorio L, Maffei L, Masullo M (2007) Soundscape in the archaeological area of Pompei. In: Proceedings of the 19th international conference on acoustics (ICA), Madrid

[CR9] Brown AL, Kang J, Gjestland T (2011). Towards standardization in soundscape preference assessment. Appl Acoust.

[CR10] Cao J, Kang J (2019). Social relationships and patterns of use in urban public spaces in China and the United Kingdom. Cities.

[CR11] Catalogue of Soundscape Intervention project, 2021, https://www.soundscape-intervention.org/ (Accessed 08.03.2022)

[CR12] Davies WJ, Mahnken PZ, Gamble P, Plack C (2009) Measuring and mapping soundscape speech intelligibility. In: Proceedings of the European conference on noise control (euronoise), Edinburgh

[CR13] De Coensel B, Botteldooren D (2008) Modeling auditory attention focusing in multisource environments. In: Proceedings of the European conference on noise control (euronoise), Acoustics’08, Paris

[CR14] Dubois D, Catherine G (2007) Cognitive evaluation of sound quality: bridging the gap between acoustic measurements and meaning. In: Proceedings of the 19th international conference on acoustics (ICA), Madrid

[CR15] Erfanian M, Mitchell A, Kang J, Aletta F (2019). The psychophysiological implications of soundscape: a systematic review of empirical literature and a research agenda. Int J Environ Res Public Health.

[CR16] EU (2002). Directive (2002/49/EC) of the European Parliament and of the council – relating to the assessment and Management of Environmental Noise.

[CR17] Fiebig A, Genuit K (2009) Development of a synthesis tool for soundscape design. In: Proceedings of the European conference on noise control (euronoise), Edinburgh

[CR18] Ge J, Hokao K (2004). Research on the sound environment of urban open space from the viewpoint of soundscape – a case study of Saga Forest park, Japan. Acta Acustica United Acustica.

[CR19] Guski R (1998) Psychological determinants of train noise annoyance. In: Proceedings of the European conference on noise control (euronoise), vol 1, Munich, pp 573–576

[CR20] Hao Y, Kang J, Krijnders JD (2015). Integrated effects of urban morphology on birdsong loudness and visibility of green areas. Landsc Urban Plan.

[CR21] Hellström B (2009) Acoustic design artefacts and methods for urban soundscapes. In: Proceedings of the 16th international congress on sound and vibration (ICSV), Krakow

[CR22] Hiramatsu K, Matsui T, Minoura K (2001) Environment similarity index concerning sonic environment toward the evaluation of sonic environment. In: Proceedings of the 17th international conference on acoustics (ICA), Rome

[CR23] Huang L, Kang J (2015). The sound environment and soundscape preservation in historic city centres - the case study of Lhasa. Environ Plann B Plann Design.

[CR24] Hume K, Ahtamad M (2009) Physiological responses and subjective estimates of soundscape elements: preliminary results for respiratory rate and EMG responses. In: Proceedings of the 38th international congress on noise control engineering, Ottawa

[CR25] Hunter MD, Eickhoff SB, Pheasant RJ, Douglas MJ, Watts GR, Farrow TFD, Hyland D, Kang J, Wilkinson ID, Horoshenkov KV, Woodruff PWR (2010). The state of tranquillity: subjective perception is shaped by contextual modulation of auditory connectivity. NeuroImage.

[CR26] International Organization for Standardization (2014) ISO 12913-1:2014 acoustics — soundscape — part 1: definition and conceptual framework, Geneva

[CR27] International Organization for Standardization (2018) ISO 12913-2:2018 acoustics — soundscape — part 2: data collection and reporting requirement, Geneva

[CR28] International Organization for Standardization. 2019. ISO 12913-3:2019 acoustics — soundscape — part 3: data analysis, Geneva

[CR29] Irwn A, Hall DA, Plack CJ (2009) How do listeners react to different urban soundscapes? An fMRI study of perception and emotion. In: Proceedings of the 38^th^ international congress on noise control engineering, Ottawa

[CR30] Järviluoma H (2000). Acoustic environments in change: five village soundscapes revisited. Soundscape: J Acoust Ecol.

[CR31] Järviluoma H, Kytö M, Truax B, Uimonen H, Vikman N, Schafer RM (2010). Acoustic environments in Change & Five Village Soundscapes.

[CR32] Jia Y, Ma H, Kang J (2020). Characteristics and evaluation of urban soundscapes worthy of preservation. J Environ Manage.

[CR33] Jia Y, Ma H, Kang J, Wang C (2020). The preservation value of urban soundscape and its determinant factors. Appl Acoust.

[CR34] Jiang L, Bristow A, Kang J, Aletta F, Thomas R, Notley H et al (2022) Ten questions concerning soundscape valuation. Build Environ 219:109231

[CR35] Jin Y, Jin H, Kang J (2020). Combined effects of the thermal-acoustic environment on subjective evaluations in urban squares. Build Environ.

[CR36] Jin Y, Jin H, Kang J (2020). Effects of sound types and sound levels on subjective environmental evaluations in different seasons. Build Environ.

[CR37] Job RFS (1988). Community response to noise: a review of factors influencing the relationship between noise exposure and reaction. J Acoust Soc Am.

[CR38] Kang J (2005). Numerical modelling of the sound fields in urban squares. J Acoust Soc Am.

[CR39] Kang J (2006). Urban sound environment.

[CR40] Kang J (2007) A systematic approach towards intentionally planning and designing soundscape in urban open public spaces. In: Proceedings of the 35th international congress on noise control engineering, Istanbul

[CR41] Kang J (2008) Urban soundscape. In: Proceedings of the Institute of Acoustics (IOA), Reading

[CR42] Kang J (2010). From understanding to designing soundscapes. Front Architect Civil Eng China.

[CR43] Kang J (2010). Soundscapes: where are we?. Proc Institute Acoust Belgium Acoust Soc.

[CR44] Kang J (2012) On the diversity of urban waterscape. In: Proceedings of the acoustics 2012 (joint meeting of the French acoustical society and UK Institute of Acoustics), Nantes, pp 3527–3532

[CR45] Kang J (2017). From dBA to soundscape indices: managing our sound environment. Front Eng Manage.

[CR46] Kang J (2018) Development in urban sound environment: towards soundscape indices. In: Proceedings of 13th Western Pacific acoustics conference, New Delhi

[CR47] Kang J (2019) Urban sound planning – a soundscape approach. In: Proceedings of the acoustics 2019, Melbourne

[CR48] Kang J (2021) Soundscape: Progress in the past 50 years and challenges in the next 50 years. In: Proceedings of the 50th international congress and exposition on noise control engineering, Washington

[CR49] Kang J, Aletta F (2020). The impact and outreach of soundscape research. Environments.

[CR50] Kang J, Aletta F, Gjestland TT, Brown LA, Botteldooren D, Schulte-Fortkamp B, Lercher P, van Kamp I, Genuit K, Fiebig A, Bento Coelho J, Maffei L, Lavia L (2016). Ten questions on the soundscapes of the built environment. Build Environ.

[CR51] Kang J, Aletta F, Margaritis E, Yang M (2018). A model for implementing soundscape maps in smart cities. Noise Map.

[CR52] Kang J, Aletta F, Oberman T, Erfanian M, Kachlicka M, Lionello M, Mitchell A (2019) Towards soundscape indices. In: Proceedings of 23rd international congress on acoustics, integrating 4th EAA Euroregio 2019, Aachen, pp 2488–2495

[CR53] Kang J, Chourmouziadou K, Sakantamis K, Wang B, Hao Y (2013). Soundscape of European cities and landscapes.

[CR54] Kang J, Hao Y, Yang M, Lavia L (2015) Soundscape evaluation and indicators for delivery sound environment. In: Proceedings of the 22nd international congress on sound and vibration, Florence

[CR55] Kang J, Schulte-Fortkamp B (2016). Soundscape and the built environment.

[CR56] Kang J, Yang W (2002) Soundscape in urban open public spaces. World Architect 144:76–79 (in Chinese)

[CR57] Kang J, Yang W, Zhang M, Nikolopoulou M (2004). Sound environment and acoustic comfort in urban spaces. Designing open spaces in the urban environment: a bioclimatic approach.

[CR58] Kihlman T (2007) Experiences of implementation of soundscapes in policies. In: Proceedings of the 36^th^ international congress on noise control engineering, Istanbul

[CR59] Lercher P (1998) Deviant dose-response curves for traffic noise in ‘sensitive areas’. In: Proceedings of the 27th international congress and exposition on noise control engineering, vol 2, Christchurch, pp 1141–1144

[CR60] Li Z, Kang J (2019). Sensitivity analysis of changes in human physiological indicators observed in soundscapes. Landsc Urban Plan.

[CR61] Licitra G, Memoli G (2005) Noise indicators and hierarchical clustering in soundscapes. In: Proceedings of the 34^th^ international congress on noise control engineering, Rio de Jeneiro

[CR62] Liu F, Liu P, Kang J, Meng Q, Wu Y, Yang D (2022). Relationships between landscape characteristics and the restorative quality of soundscapes in urban blue spaces. Appl Acoust.

[CR63] Liu J, Kang J, Behm H, Luo T (2014). Effects of landscape on soundscape perception: soundwalks in city parks. Landsc Urban Plan.

[CR64] Mao L, Kang J, Jin H (2013). Acoustic characteristics of ethnic Miao and Hans traditional settlement spaces in Guizhou province. Architect J.

[CR65] Margaritis E, Kang J (2017). Relationship between green space-related morphology and noise pollution. Ecol Indic.

[CR66] Margaritis E, Kang J (2017). Soundscape mapping in environmental noise management and urban planning: case studies in two UK cities. Noise Map.

[CR67] Margaritis E, Kang J, Filipan K, Botteldooren D (2018). The influence of vegetation and surrounding traffic noise parameters on the sound environment in urban parks. Appl Geogr.

[CR68] McGinley R (2005) Stockholm sound sanctuaries: a public sound art project. In: Proceedings of the 12th international congress on sound and vibration (ICSV), Lisbon

[CR69] Medvedev O, Shepherd D, Hautus M (2015). The restorative potential of soundscapes: a physiological investigation. Appl Acoust.

[CR70] Mitchell A, Oberman T, Aletta F, Erfanian M, Kachlicka M, Lionello M, Kang J (2020). The soundscape indices (SSID) protocol: a method for urban soundscape surveys—questionnaires with acoustical and contextual information. Appl Sci.

[CR71] Niessen ME, Krijnders D, Andringa TC (2009) Understanding a soundscape through its components. In: Proceedings of the European conference on noise control (euronoise), Edinburgh

[CR72] Noise Capture, 2022. https://noise-planet.org/noisecapture.html (Accessed 03.05.2022)

[CR73] Pheasant RJ, Watts GR, Horoshenkov KV, Barrett BT (2009). The acoustic and visual factors influencing the construction of tranquil space in urban and rural environments: tranquil spaces – quiet places?. J Acoust Soc Am.

[CR74] Qin Y, Zhao W, Kang J (2020). Effects of background sound sources types on emotion and activities in activity spaces of elderly care facilities. J Hum Settlements West China.

[CR75] Raimbault M, Bérengier M, Dubois D (2001) Common factors in the identification of urban soundscapes: pilot studies in two French cities: Lyon and Nantes. In: Proceedings of the 17th international conference on acoustics (ICA), Rome

[CR76] Ren X, Kang J (2015). Interactions between landscape elements and tranquillity evaluation based on eye tracking experiments. J Acoust Soc Am.

[CR77] Ren X, Kang J, Zhu P, Wang S (2018). Effects of soundscape on rural landscape evaluations. Environ Impact Assess Rev.

[CR78] Ren X, Kang J, Zhu P, Wang S (2018). Soundscape expectations of rural tourism: a comparison between Chinese and English potential tourists. J Acoust Soc Am.

[CR79] Schafer RM (1977). The tuning of the world.

[CR80] Schomer P, Brown L, De Coensel B, Genuit K, Gjestland T, Jeon JY, Kang J, Newman P, Schulte-Fortkamp B, Watts GR (2010) On efforts to standardize a graphical description of the soundscape concept. In: Proceedings of the 39^th^ international congress on noise control engineering, Lisbon

[CR81] Shepherd C, Grimwood C (2009) Sound evaluation: the development of the concept of quiet zones within the City of London. In: Proceedings of the European conference on noise control (euronoise), Edinburgh

[CR82] Smyrnova Y, Kang J (2010). Determination of perceptual auditory attributes for the auralization of urban soundscapes. Noise Control Engineering Journal.

[CR83] Sounds of New York City project, 2014. https://wp.nyu.edu/sonyc/ (Accessed 08.03.2022)

[CR84] Tong H, Aletta F, Mitchell A, Oberman T, Kang J (2021). Increases in noise complaints during the COVID-19 lockdown in spring 2020: a case study in greater London, UK. Sci Total Environ.

[CR85] Torresin S, Albatici R, Aletta F, Babich F, Oberman T, Siboni S, Kang J (2020). Indoor soundscape assessment: a principal components model of acoustic perception in residential buildings. Build Environ.

[CR86] UK DEFRA, 2010. https://assets.publishing.service.gov.uk/government/uploads/system/uploads/attachment_data/file/69533/pb13750-noise-policy.pdf (Accessed 08.03.2022)

[CR87] Urban Soundscapes of the World project, 2017, http://urban-soundscapes.org/ (Accessed 08.03.2022)

[CR88] Watts GR, Hunter MD, Douglas M, Pheasant RJ, Farrow TFD, Wilkinson ID, Kang J, Horoshenkov K, Woodruff PW (2009) The use of fMRI techniques to investigate the perception of tranquility. In: Proceedings of the 38^th^ international congress on noise control engineering, Ottawa

[CR89] Welsh Government, 2018, https://gov.wales/noise-and-soundscape-action-plan-2018-2023-0 (Accessed 08.03.2022)

[CR90] Woloszyn P, Leduc T, Joanne P (2009) Urban soundmarks psychophysical geodimensioning: towards ambient pointers geosystemic computation. In: Proceedings of the European conference on noise control (euronoise), Edinburgh

[CR91] Xiao J, Lavia L, Kang J (2017). Towards a participatory urban soundscape planning framework. J Environ Plan Manag.

[CR92] Xu C, Kang J (2019). Soundscape evaluation: binaural or monaural?. J Acoust Soc Am.

[CR93] Yang W, Kang J (2005). Acoustic comfort evaluation in urban open public spaces. Appl Acoust.

[CR94] Yang W, Kang J (2005). Soundscape and sound preferences in urban squares. J Urban Des.

[CR95] Yu C, Kang J (2014). Soundscape in the sustainable living environment: a cross-cultural comparison between the UK and Taiwan. Sci Total Environ.

[CR96] Yu L, Kang J (2008). Effects of social, demographic and behavioral factors on sound level evaluation in urban open spaces. J Acoust Soc Am.

[CR97] Yu L, Kang J (2009). Modeling subjective evaluation of soundscape quality in urban open spaces – an artificial neural network approach. J Acoust Soc Am.

[CR98] Yu W, Kang J (2018). Resistance of villages to elevated-road traffic noise. J Environ Plan Manag.

[CR99] Zhang D, Zhang M, Liu D, Kang J (2016). Soundscape evaluation in Han Chinese Buddhist temples. Appl Acoust.

[CR100] Zhang M, Kang J (2007). Towards the evaluation, description and creation of soundscape in urban open spaces. Environ Plann B: Plann Des.

